# Supplementary material for: Genome-Wide Association Study Reveals the Genetic Basis of Duck Plumage Colors
Source: Genes (Basel). 2023 Mar 31;14(4):856. doi: 10.3390/genes14040856 (PMC10137861; doi:10.3390/genes14040856)
Supplement: Supplementary file 1 [file genes-14-00856-s001.zip › genes-2247379-supplementary.pdf]

SUPPLEMENTARY INFORMATION

Table S1. Genome-wide SNPs associated with black plumage color trait

SNP ID	Chromosome	Position	P-value	Gene
1:31342484	1	31342484	4.08E-09	NELL2
1:31346687	1	31346687	2.54E-09	NELL2
1:53516478	1	53516478	6.56E-10	CARD10
1:94865544	1	94865544	5.84E-10	PTGFRN
1:94868866	1	94868866	4.23E-09	PTGFRN
1:136032644	1	136032644	5.64E-13	NA
1:136032650	1	136032650	3.88E-13	NA
1:136032653	1	136032653	3.88E-13	NA
1:136032657	1	136032657	3.31E-12	NA
1:136032659	1	136032659	3.88E-13	NA
1:136032667	1	136032667	1.07E-15	NA
1:147626207	1	147626207	4.41E-12	NA
1:147629767	1	147629767	2.07E-09	NA
1:147630149	1	147630149	6.01E-11	NA
1:147630151	1	147630151	6.01E-11	NA
1:147630154	1	147630154	6.01E-11	NA
2:67044268	2	67044268	6.88E-10	NA
3:23231760	3	23231760	1.51E-09	NA
3:55495295	3	55495295	4.09E-09	NA
3:55495296	3	55495296	3.33E-09	NA
3:69817655	3	69817655	9.72E-10	NA
3:69817865	3	69817865	1.19E-09	NA
3:69817890	3	69817890	5.59E-10	NA
3:74873152	3	74873152	2.16E-09	NA
3:74873769	3	74873769	2.61E-10	NA
3:74875382	3	74875382	9.75E-11	NA
3:74875501	3	74875501	8.55E-12	NA
3:74876367	3	74876367	5.09E-12	NA
3:90536882	3	90536882	9.47E-10	NA
3:98873682	3	98873682	1.54E-09	AKIRIN2
3:107628359	3	107628359	9.37E-11	NA
4:21614761	4	21614761	3.87E-09	NA
4:21617220	4	21617220	4.51E-11	NA
4:21660201	4	21660201	4.09E-09	NA
4:21670809	4	21670809	5.57E-10	NA
4:21766289	4	21766289	1.33E-09	NA
4:21766320	4	21766320	1.47E-09	NA
5:25376130	5	25376130	1.58E-09	RYR3

5:25493233	5	25493233	2.78E-09	RYR3
5:25530667	5	25530667	4.40E-09	RYR3
5:25548395	5	25548395	2.58E-09	RYR3
5:33115002	5	33115002	3.02E-09	NA
7:1487809	7	1487809	5.73E-10	PGAP1
7:1489779	7	1489779	4.15E-09	PGAP1
7:1489969	7	1489969	3.02E-10	PGAP1
7:1490108	7	1490108	1.71E-09	PGAP1
7:1490744	7	1490744	1.65E-09	PGAP1
7:1495601	7	1495601	2.57E-09	PGAP1
7:1496142	7	1496142	9.06E-10	PGAP1
7:1497045	7	1497045	1.08E-11	PGAP1
8:8294483	8	8294483	3.77E-09	NA
10:10022869	10	10022869	2.12E-09	FRMPD3
12:20213249	12	20213249	6.15E-10	TCF25
12:20213250	12	20213250	6.15E-10	TCF25
12:20222793	12	20222793	8.46E-21	MC1R
12:20223117	12	20223117	1.12E-25	MC1R
13:14082768	13	14082768	2.77E-09	NA
13:15933623	13	15933623	1.98E-09	NA
18:7771795	18	7771795	1.51E-09	USP20
22:6749040	22	6749040	3.38E-09	NA
22:6751763	22	6751763	2.92E-10	NA
22:6752227	22	6752227	2.21E-10	NA
25:3735640	25	3735640	3.33E-10	NA
27:1186253	27	1186253	1.37E-09	NA

Table S2. Genome-wide SNPs associated with white plumage color trait

SNP ID	Chromosome	Position	P- value	Gene
1:67989812	1	67989812	2.85E-10	NA
1:68755848	1	68755848	2.28E-10	NA
1:69041641	1	69041641	1.86E-09	NA
1:69834116	1	69834116	1.37E-11	NA
1:70468366	1	70468366	3.11E-09	WNT7B
1:73405198	1	73405198	1.13E-10	NA
1:73591785	1	73591785	5.32E-12	NA
1:74192281	1	74192281	2.93E-10	NA
1:74594486	1	74594486	4.38E-11	NA
1:76447822	1	76447822	2.79E-09	NA
1:76614118	1	76614118	7.85E-13	NA
1:76804165	1	76804165	5.84E-11	VWF
1:76873212	1	76873212	1.54E-09	NA
1:140992689	1	140992689	4.80E-10	MTUS2

2:20539680	2	20539680	5.76E-12	CACNB2
2:74953555	2	74953555	9.90E-10	NA
2:103327114	2	103327114	2.10E-09	NA
2:103396008	2	103396008	9.68E-10	NA
2:103396037	2	103396037	7.46E-10	NA
2:103396305	2	103396305	6.38E-10	NA
2:103420257	2	103420257	2.21E-09	NA
2:103429350	2	103429350	2.55E-09	NA
3:1977736	3	1977736	8.39E-11	NA
3:97597676	3	97597676	5.97E-09	NA
3:97598088	3	97598088	5.07E-09	NA
3:97599256	3	97599256	5.63E-09	NA
3:97618584	3	97618584	5.19E-09	CEP162
3:97619994	3	97619994	7.07E-10	CEP162
3:97623229	3	97623229	1.40E-10	CEP162
3:97625729	3	97625729	2.47E-09	CEP162
3:97626048	3	97626048	5.61E-09	CEP162
3:97626066	3	97626066	3.21E-10	CEP162
3:97626129	3	97626129	6.82E-11	CEP162
3:97632975	3	97632975	8.62E-10	CEP162
3:97637880	3	97637880	1.16E-10	CEP162
3:97637901	3	97637901	3.76E-09	CEP162
3:97641052	3	97641052	1.59E-11	CEP162
3:97645677	3	97645677	9.21E-11	CEP162
3:97651131	3	97651131	1.14E-09	CEP162
3:97654937	3	97654937	1.28E-11	CEP162
3:97656911	3	97656911	1.76E-09	CEP162
3:97657482	3	97657482	4.41E-09	CEP162
3:97659302	3	97659302	4.92E-14	NA
3:97706013	3	97706013	5.15E-10	NA
3:97708190	3	97708190	1.35E-09	NA
3:97708203	3	97708203	3.19E-11	NA
3:97716547	3	97716547	2.70E-15	NA
3:97716626	3	97716626	1.62E-15	NA
3:97718460	3	97718460	9.26E-11	NA
3:97719694	3	97719694	4.13E-13	NA
3:97719759	3	97719759	3.84E-10	NA
3:97724332	3	97724332	1.01E-11	NA
3:97729877	3	97729877	4.01E-09	NA
3:97733818	3	97733818	1.32E-18	NA
3:97734178	3	97734178	1.24E-16	NA
3:97735001	3	97735001	5.56E-11	NA
3:97735865	3	97735865	7.45E-13	NA
3:97737410	3	97737410	7.15E-13	NA

3:97737474	3	97737474	2.02E-09	NA
3:97739237	3	97739237	3.88E-09	NA
3:97740069	3	97740069	1.69E-11	NA
3:97746578	3	97746578	3.34E-12	NA
3:97755710	3	97755710	2.65E-15	NA
3:97756751	3	97756751	4.43E-11	NA
3:97758294	3	97758294	2.97E-17	NA
3:97758438	3	97758438	9.29E-18	NA
3:97758483	3	97758483	4.61E-15	NA
3:97758930	3	97758930	6.52E-10	NA
3:97761828	3	97761828	2.10E-09	NA
3:97762688	3	97762688	1.46E-11	NA
3:97763477	3	97763477	6.73E-11	NA
3:97763874	3	97763874	8.62E-10	NA
3:97763880	3	97763880	8.62E-10	NA
3:97764222	3	97764222	1.50E-10	NA
3:97764302	3	97764302	1.71E-11	NA
3:97764315	3	97764315	4.03E-09	NA
3:97764404	3	97764404	2.23E-10	NA
3:97764670	3	97764670	2.96E-13	NA
3:97764891	3	97764891	3.49E-11	NA
3:97765069	3	97765069	1.19E-13	NA
3:97767279	3	97767279	6.44E-16	NA
3:97768697	3	97768697	5.88E-14	NA
3:97768785	3	97768785	4.52E-10	NA
3:97769868	3	97769868	2.43E-12	NA
3:97773306	3	97773306	6.53E-13	NA
3:97774157	3	97774157	2.81E-11	NA
3:97774223	3	97774223	7.54E-16	NA
3:97775680	3	97775680	2.12E-11	NA
3:98055569	3	98055569	3.09E-10	NA
3:98055571	3	98055571	3.09E-10	NA
3:98081563	3	98081563	2.44E-09	NA
3:98092395	3	98092395	2.53E-10	NA
3:98131927	3	98131927	6.30E-11	NA
3:113303742	3	113303742	6.45E-10	PKHD1
3:113305763	3	113305763	1.41E-11	PKHD1
3:113307629	3	113307629	1.39E-13	PKHD1
3:113307650	3	113307650	5.21E-15	PKHD1
3:113307830	3	113307830	4.39E-09	PKHD1
3:113312999	3	113312999	1.38E-10	PKHD1
3:113313962	3	113313962	1.29E-09	PKHD1
3:113314425	3	113314425	3.33E-09	PKHD1
3:113315920	3	113315920	3.82E-10	PKHD1

3:113327709	3	113327709	8.86E-10	PKHD1
3:113333806	3	113333806	4.50E-09	PKHD1
3:113359406	3	113359406	1.31E-12	PKHD1
3:113359980	3	113359980	5.76E-09	PKHD1
3:113368805	3	113368805	8.48E-10	PKHD1
3:113368828	3	113368828	1.40E-09	PKHD1
3:113368834	3	113368834	1.40E-09	PKHD1
3:113375100	3	113375100	1.20E-09	PKHD1
3:113375104	3	113375104	1.20E-09	PKHD1
3:113382936	3	113382936	5.07E-09	PKHD1
3:113386535	3	113386535	2.47E-09	PKHD1
3:113401659	3	113401659	1.70E-09	PKHD1
4:23106802	4	23106802	1.00E-09	NA
4:33958344	4	33958344	1.30E-09	NA
4:33980544	4	33980544	1.05E-10	SLIT2
4:34070699	4	34070699	4.80E-09	SLIT2
4:35725648	4	35725648	9.17E-10	NA
4:62404125	4	62404125	4.74E-09	NA
4:62821084	4	62821084	4.74E-09	NA
4:63027059	4	63027059	1.14E-11	PDGFC
4:63239857	4	63239857	9.74E-10	GRIA2
4:65599955	4	65599955	8.10E-10	NA
5:33447645	5	33447645	1.77E-09	NA
5:40339047	5	40339047	3.61E-09	DLST
5:40339181	5	40339181	1.70E-11	DLST
5:40339281	5	40339281	4.64E-10	DLST
5:40339798	5	40339798	3.16E-11	DLST
5:40341539	5	40341539	3.10E-11	DLST
5:40341556	5	40341556	3.10E-11	DLST
5:40341969	5	40341969	4.84E-09	DLST
5:40341980	5	40341980	4.84E-09	DLST
5:40341996	5	40341996	3.37E-10	DLST
5:40342044	5	40342044	9.36E-13	DLST
5:40342060	5	40342060	1.98E-13	DLST
5:40342102	5	40342102	1.05E-09	DLST
5:40342551	5	40342551	1.57E-10	DLST
5:40342730	5	40342730	1.38E-09	DLST
5:40343377	5	40343377	2.52E-12	DLST
5:40736197	5	40736197	2.94E-09	NA
5:40740823	5	40740823	5.65E-09	NA
5:40746009	5	40746009	1.73E-09	NA
5:40746515	5	40746515	1.70E-09	NA
5:40749063	5	40749063	8.82E-11	NA
5:40750286	5	40750286	1.18E-22	NA

5:40750296	5	40750296	1.18E-22	NA
5:40750530	5	40750530	1.83E-15	NA
5:40750641	5	40750641	3.84E-11	NA
5:40758393	5	40758393	1.75E-15	NA
5:40758489	5	40758489	3.67E-13	NA
5:40758492	5	40758492	3.67E-13	NA
5:40758650	5	40758650	4.95E-12	NA
5:40759084	5	40759084	8.89E-12	NA
5:40759459	5	40759459	3.75E-11	NA
5:40760234	5	40760234	8.27E-10	NA
5:40760276	5	40760276	1.19E-09	NA
5:40760732	5	40760732	1.30E-12	NA
5:40761156	5	40761156	5.91E-21	NA
5:40761617	5	40761617	7.03E-18	NA
5:40762245	5	40762245	7.29E-10	NA
5:40763173	5	40763173	2.06E-15	NA
5:40763537	5	40763537	6.93E-13	NA
5:40763591	5	40763591	1.31E-09	NA
5:40763598	5	40763598	2.54E-09	NA
5:40763693	5	40763693	2.51E-14	NA
5:40763702	5	40763702	2.51E-14	NA
5:40763723	5	40763723	9.67E-15	NA
5:40765372	5	40765372	2.33E-09	NA
5:40766699	5	40766699	2.84E-09	NA
5:40767104	5	40767104	2.06E-11	NA
5:40768315	5	40768315	9.33E-17	NA
5:40768573	5	40768573	2.31E-16	NA
5:40768760	5	40768760	1.87E-13	NA
5:40770343	5	40770343	8.47E-14	NA
5:40771044	5	40771044	2.53E-10	NA
5:40772477	5	40772477	2.69E-09	NA
5:40773796	5	40773796	3.66E-10	NA
5:40775592	5	40775592	2.92E-11	NA
5:40775679	5	40775679	6.51E-16	NA
5:40776116	5	40776116	4.26E-15	NA
5:40776299	5	40776299	8.43E-14	NA
5:40778024	5	40778024	1.25E-11	NA
5:40779327	5	40779327	5.57E-09	NA
5:40780383	5	40780383	4.08E-10	NA
5:40780403	5	40780403	4.89E-11	NA
5:40782087	5	40782087	1.10E-10	NA
5:40782385	5	40782385	2.68E-10	NA
5:40783925	5	40783925	1.69E-13	NA
5:40784035	5	40784035	8.90E-11	NA

5:40784116	5	40784116	1.38E-09	NA
5:40784128	5	40784128	1.38E-09	NA
5:40784129	5	40784129	1.38E-09	NA
6:1899118	6	1899118	1.38E-09	NA
6:2009654	6	2009654	1.82E-09	NA
6:2014655	6	2014655	3.66E-11	NA
6:2014709	6	2014709	1.30E-11	NA
6:2017709	6	2017709	3.30E-14	NA
6:2020830	6	2020830	2.43E-09	NA
6:16592716	6	16592716	4.99E-09	HERC4
7:18342844	7	18342844	9.29E-11	SLC49A4
7:18343709	7	18343709	1.04E-12	SLC49A4
7:18344243	7	18344243	1.49E-15	SLC49A4
7:18359372	7	18359372	4.39E-10	SLC49A4
7:18360457	7	18360457	2.74E-10	SLC49A4
7:18360481	7	18360481	1.34E-09	SLC49A4
7:18360492	7	18360492	6.91E-10	SLC49A4
7:18361077	7	18361077	4.60E-09	SLC49A4
7:18361112	7	18361112	1.93E-09	SLC49A4
7:18374006	7	18374006	4.21E-09	NA
7:18374028	7	18374028	5.88E-10	NA
7:18374086	7	18374086	6.90E-11	NA
7:18374091	7	18374091	1.21E-10	NA
7:18374114	7	18374114	3.17E-09	NA
7:18374253	7	18374253	3.00E-10	NA
7:18374940	7	18374940	7.68E-10	NA
7:18380469	7	18380469	3.24E-13	NA
7:18430362	7	18430362	1.17E-09	SEMA5B
7:18430412	7	18430412	1.19E-09	SEMA5B
7:18430574	7	18430574	2.66E-10	SEMA5B
7:18430711	7	18430711	9.29E-10	SEMA5B
7:18430732	7	18430732	9.23E-11	SEMA5B
7:18430776	7	18430776	3.03E-11	SEMA5B
7:18431104	7	18431104	4.41E-11	SEMA5B
7:18431372	7	18431372	8.95E-12	SEMA5B
7:18431413	7	18431413	3.55E-11	SEMA5B
7:18431710	7	18431710	5.51E-10	SEMA5B
7:18431751	7	18431751	1.89E-09	SEMA5B
7:18434231	7	18434231	1.42E-09	SEMA5B
7:18438781	7	18438781	4.80E-09	SEMA5B
7:18443860	7	18443860	1.82E-11	SEMA5B
7:18445525	7	18445525	4.52E-10	SEMA5B
7:18453106	7	18453106	1.66E-13	SEMA5B
7:18477662	7	18477662	4.34E-09	SEMA5B

7:18477663	7	18477663	4.34E-09	SEMA5B
7:18478141	7	18478141	2.35E-10	SEMA5B
7:18478149	7	18478149	2.79E-10	SEMA5B
7:18482590	7	18482590	8.51E-13	SEMA5B
7:18483486	7	18483486	3.33E-13	SEMA5B
7:18483708	7	18483708	7.36E-14	SEMA5B
7:18484740	7	18484740	2.85E-09	SEMA5B
7:18485845	7	18485845	1.06E-13	SEMA5B
7:18486098	7	18486098	2.11E-16	SEMA5B
7:18486103	7	18486103	6.90E-16	SEMA5B
7:18488139	7	18488139	4.69E-09	SEMA5B
7:18489070	7	18489070	3.90E-12	SEMA5B
7:18499478	7	18499478	5.19E-09	SEMA5B
7:18499479	7	18499479	3.89E-09	SEMA5B
7:18500837	7	18500837	6.16E-13	NA
7:18500838	7	18500838	6.16E-13	NA
7:18505056	7	18505056	2.03E-10	NA
7:18537129	7	18537129	8.45E-13	NA
7:18537533	7	18537533	2.73E-11	NA
7:18615490	7	18615490	1.41E-12	NA
7:18624738	7	18624738	2.60E-09	NA
7:18630491	7	18630491	8.25E-12	NA
7:18631502	7	18631502	5.60E-09	NA
7:18635638	7	18635638	2.59E-11	NA
7:18643043	7	18643043	7.14E-12	NA
7:18648606	7	18648606	6.37E-10	NA
7:27886962	7	27886962	1.02E-10	NA
7:27894350	7	27894350	3.45E-09	NA
7:27897265	7	27897265	5.14E-11	NA
7:27934239	7	27934239	4.96E-15	NA
7:27950812	7	27950812	4.86E-09	ZNF385B
7:28022066	7	28022066	3.35E-09	ZNF385B
7:28028059	7	28028059	4.02E-09	ZNF385B
7:28090593	7	28090593	1.50E-09	ZNF385B
7:33030200	7	33030200	5.15E-09	ACVR2A
7:33030202	7	33030202	5.15E-09	ACVR2A
7:33030206	7	33030206	5.15E-09	ACVR2A
7:33030210	7	33030210	5.15E-09	ACVR2A
8:25582988	8	25582988	8.85E-12	NA
8:28180119	8	28180119	3.51E-09	NA
8:28181078	8	28181078	7.02E-10	NA
8:28182195	8	28182195	4.35E-10	NA
8:28500750	8	28500750	2.08E-14	NA
9:5336598	9	5336598	2.16E-09	LPP

9:5479241	9	5479241	8.11E-10	NA
9:5479248	9	5479248	8.11E-10	NA
11:18490095	11	18490095	4.36E-09	PCSK6
12:8118077	12	8118077	3.38E-09	ADCY7
12:11414449	12	11414449	7.00E-13	NA
12:11429694	12	11429694	1.70E-09	NA
12:11437838	12	11437838	9.72E-14	GPI
12:11439703	12	11439703	8.06E-11	GPI
12:11443345	12	11443345	1.44E-11	GPI
12:11454713	12	11454713	3.25E-11	NA
12:11458763	12	11458763	6.88E-11	NA
12:11462280	12	11462280	6.73E-12	NA
12:11466191	12	11466191	4.23E-09	NA
12:11468042	12	11468042	2.32E-12	NA
12:11471103	12	11471103	4.39E-11	NA
12:11482925	12	11482925	7.03E-11	NA
12:11485882	12	11485882	5.14E-13	NA
12:11497520	12	11497520	1.61E-14	NA
12:11507779	12	11507779	1.33E-09	NA
12:11512281	12	11512281	3.21E-11	NA
12:11527234	12	11527234	1.01E-09	WTIP
12:11533004	12	11533004	4.25E-11	WTIP
12:11538584	12	11538584	8.82E-10	WTIP
12:11538613	12	11538613	1.20E-09	WTIP
12:11539010	12	11539010	1.51E-09	WTIP
12:11539391	12	11539391	8.42E-13	WTIP
12:11539711	12	11539711	2.81E-10	WTIP
12:11546332	12	11546332	2.02E-11	WTIP
12:11549780	12	11549780	3.87E-09	WTIP
12:11554279	12	11554279	1.88E-11	WTIP
12:11556056	12	11556056	1.91E-10	WTIP
12:11557704	12	11557704	4.52E-09	WTIP
12:11560635	12	11560635	3.13E-10	WTIP
12:11561718	12	11561718	7.43E-11	WTIP
12:11562441	12	11562441	1.65E-14	WTIP
12:11564021	12	11564021	2.18E-11	WTIP
12:11567578	12	11567578	7.22E-13	WTIP
12:11569730	12	11569730	1.41E-09	WTIP
12:11569908	12	11569908	8.59E-13	WTIP
12:11570971	12	11570971	1.68E-11	WTIP
12:11571905	12	11571905	6.06E-12	WTIP
12:11573132	12	11573132	1.17E-09	WTIP
12:11573395	12	11573395	9.33E-13	WTIP
12:11573738	12	11573738	2.16E-09	WTIP

12:11575479	12	11575479	6.69E-11	WTIP
12:11576711	12	11576711	3.25E-11	WTIP
12:11577157	12	11577157	2.23E-11	WTIP
13:7443936	13	7443936	2.21E-09	ITIH4
13:7538806	13	7538806	2.89E-09	NA
13:9704572	13	9704572	2.10E-13	NA
13:10134372	13	10134372	3.28E-09	MGLL
13:10146103	13	10146103	8.48E-14	MGLL
13:11847388	13	11847388	2.22E-09	NA
13:11847389	13	11847389	2.22E-09	NA
13:11847392	13	11847392	2.06E-10	NA
13:11847393	13	11847393	2.06E-10	NA
13:11847403	13	11847403	2.34E-09	NA
13:11950016	13	11950016	2.33E-09	NA
13:11954874	13	11954874	2.42E-09	NA
13:11954883	13	11954883	1.68E-10	NA
13:11975819	13	11975819	2.07E-10	NA
13:11980770	13	11980770	3.82E-20	ARL8B
13:11982812	13	11982812	1.59E-12	ARL8B
13:11982818	13	11982818	1.59E-12	ARL8B
13:11982822	13	11982822	1.59E-12	ARL8B
13:11982827	13	11982827	1.59E-12	ARL8B
13:11982878	13	11982878	9.19E-52	ARL8B
13:11982968	13	11982968	8.57E-11	ARL8B
13:11983006	13	11983006	4.48E-16	ARL8B
13:11985159	13	11985159	6.82E-14	ARL8B
13:11985432	13	11985432	2.73E-11	ARL8B
13:11985865	13	11985865	2.40E-12	ARL8B
13:11985927	13	11985927	5.97E-09	ARL8B
13:11985938	13	11985938	2.10E-13	ARL8B
13:11986165	13	11986165	2.03E-10	ARL8B
13:11986442	13	11986442	3.26E-52	ARL8B
13:11986592	13	11986592	1.74E-12	ARL8B
13:11986601	13	11986601	8.82E-12	ARL8B
13:11986689	13	11986689	1.26E-11	ARL8B
13:11988714	13	11988714	4.23E-49	ARL8B
13:11989419	13	11989419	1.53E-13	ARL8B
13:11991274	13	11991274	4.89E-11	ARL8B
13:11991275	13	11991275	1.76E-12	ARL8B
13:11991283	13	11991283	1.45E-10	ARL8B
13:11991291	13	11991291	2.24E-14	ARL8B
13:12044219	13	12044219	2.73E-09	NA
13:12044361	13	12044361	6.60E-10	NA
13:12044780	13	12044780	1.35E-10	NA

13:12131521	13	12131521	1.12E-09	ITPR1
13:12131739	13	12131739	3.79E-13	ITPR1
13:12132015	13	12132015	6.01E-16	ITPR1
13:12132145	13	12132145	1.80E-11	ITPR1
13:12132187	13	12132187	1.91E-17	ITPR1
13:12132233	13	12132233	1.72E-19	ITPR1
13:12132285	13	12132285	3.29E-12	ITPR1
13:12147574	13	12147574	2.76E-19	ITPR1
13:12156389	13	12156389	2.50E-11	ITPR1
13:12156704	13	12156704	3.87E-10	ITPR1
13:13300326	13	13300326	8.45E-10	NA
13:13309261	13	13309261	1.13E-09	NA
13:13325004	13	13325004	2.29E-09	NA
13:14289145	13	14289145	2.44E-10	PPP4R2
13:14289159	13	14289159	2.44E-10	PPP4R2
13:14289163	13	14289163	4.52E-10	PPP4R2
13:15399026	13	15399026	5.56E-58	MITF
13:15399414	13	15399414	0.00E+00	MITF
13:15399439	13	15399439	0.00E+00	MITF
13:15399625	13	15399625	1.81E-66	MITF
13:15399727	13	15399727	5.41E-55	MITF
13:15399791	13	15399791	1.70E-77	MITF
13:15399795	13	15399795	1.70E-77	MITF
13:15399953	13	15399953	9.40E-24	MITF
13:15399955	13	15399955	2.22E-32	MITF
13:15400127	13	15400127	1.70E-77	MITF
13:15400396	13	15400396	2.01E-82	MITF
13:15402575	13	15402575	1.57E-25	MITF
13:15403282	13	15403282	4.80E-60	MITF
13:15404023	13	15404023	7.39E-63	MITF
13:15404980	13	15404980	5.56E-58	MITF
13:15411160	13	15411160	5.60E-10	MITF
13:15411658	13	15411658	6.52E-63	MITF
13:15411837	13	15411837	7.03E-14	MITF
13:15412000	13	15412000	2.38E-14	MITF
13:15412425	13	15412425	2.68E-22	MITF
13:15412428	13	15412428	2.79E-10	MITF
13:15412570	13	15412570	1.06E-50	MITF
13:15412592	13	15412592	2.31E-39	MITF
13:15412878	13	15412878	3.95E-21	MITF
13:15412950	13	15412950	7.34E-23	MITF
13:15412967	13	15412967	4.11E-12	MITF
13:15413071	13	15413071	9.31E-30	MITF
13:15413199	13	15413199	2.11E-42	MITF

13:15413219	13	15413219	2.11E-42	MITF
13:15413509	13	15413509	3.78E-26	MITF
13:15413626	13	15413626	1.61E-14	MITF
13:15413700	13	15413700	3.69E-17	MITF
13:15413811	13	15413811	1.06E-50	MITF
13:15413853	13	15413853	9.35E-23	MITF
13:15414262	13	15414262	1.87E-62	MITF
13:15414267	13	15414267	1.87E-62	MITF
13:15415825	13	15415825	1.95E-19	MITF
13:15416145	13	15416145	1.68E-36	MITF
13:15417373	13	15417373	2.18E-28	MITF
13:15417813	13	15417813	5.56E-58	MITF
13:15417918	13	15417918	2.65E-41	MITF
13:15418049	13	15418049	7.76E-34	MITF
13:15418199	13	15418199	6.56E-12	MITF
13:15418646	13	15418646	2.83E-22	MITF
13:15418785	13	15418785	3.14E-43	MITF
13:15419385	13	15419385	7.39E-63	MITF
13:15421039	13	15421039	2.63E-26	MITF
13:15421572	13	15421572	5.31E-19	MITF
13:15421679	13	15421679	2.90E-39	MITF
13:15421717	13	15421717	1.40E-13	MITF
13:15421903	13	15421903	2.69E-11	MITF
13:15421983	13	15421983	9.41E-13	MITF
13:15422029	13	15422029	5.49E-38	MITF
13:15424125	13	15424125	4.45E-29	MITF
13:15425177	13	15425177	2.25E-45	MITF
13:15426452	13	15426452	4.33E-11	MITF
13:15491773	13	15491773	1.05E-09	NA
13:15499802	13	15499802	6.65E-10	NA
13:15520054	13	15520054	3.40E-09	NA
13:15520770	13	15520770	7.86E-10	NA
13:16668662	13	16668662	9.45E-10	SLC25A26
13:16671127	13	16671127	2.78E-10	SLC25A26
13:16991098	13	16991098	2.95E-10	MAGI1
13:18266118	13	18266118	1.11E-11	PTPRG
13:18266119	13	18266119	1.11E-11	PTPRG
13:18283850	13	18283850	5.81E-09	PTPRG
13:18308347	13	18308347	5.89E-10	PTPRG
13:18308395	13	18308395	1.05E-09	PTPRG
13:18313164	13	18313164	1.36E-09	PTPRG
13:18325053	13	18325053	1.22E-09	PTPRG
13:18329396	13	18329396	8.50E-10	PTPRG
13:18338031	13	18338031	5.68E-09	PTPRG

13:18344145	13	18344145	5.96E-09	PTPRG
13:18346733	13	18346733	2.76E-10	PTPRG
13:18356239	13	18356239	4.32E-09	PTPRG
13:18356933	13	18356933	3.09E-09	PTPRG
13:18365497	13	18365497	4.01E-09	PTPRG
13:18366825	13	18366825	1.72E-09	PTPRG
13:18375126	13	18375126	4.81E-09	PTPRG
13:18376495	13	18376495	2.64E-09	PTPRG
13:18383953	13	18383953	6.01E-09	PTPRG
13:18391936	13	18391936	4.93E-10	PTPRG
13:18413222	13	18413222	1.66E-10	PTPRG
13:18413672	13	18413672	1.81E-09	PTPRG
13:18416582	13	18416582	7.33E-10	PTPRG
13:18416630	13	18416630	6.28E-10	PTPRG
13:18418084	13	18418084	2.72E-10	PTPRG
13:19733009	13	19733009	6.82E-10	QRICH1
13:19733037	13	19733037	5.51E-10	QRICH1
13:20472421	13	20472421	3.04E-09	NA
14:17934715	14	17934715	2.71E-09	ARHGAP26
16:5522302	16	5522302	3.12E-09	NA
18:6001070	18	6001070	6.21E-10	NA
18:7586264	18	7586264	9.24E-11	NA
18:9162010	18	9162010	4.66E-09	NA
18:11239371	18	11239371	3.22E-09	MVB12B
18:11240147	18	11240147	2.67E-09	MVB12B
18:11246457	18	11246457	4.13E-09	NA
18:11256062	18	11256062	1.77E-09	NA
18:11259658	18	11259658	8.79E-10	NA
18:11269611	18	11269611	2.87E-09	NA
18:11269842	18	11269842	4.29E-09	NA
18:11296444	18	11296444	1.86E-09	NA
18:11296926	18	11296926	2.22E-13	NA
18:11317379	18	11317379	1.70E-10	NA
19:3099743	19	3099743	1.37E-09	NA
19:12195339	19	12195339	1.25E-09	NA
29:1994431	29	1994431	2.99E-10	NA
29:2423462	29	2423462	3.10E-09	NA
